# Yeast as a model system to study metabolic impact of selenium compounds

**DOI:** 10.15698/mic2015.05.200

**Published:** 2015-04-08

**Authors:** Enrique Herrero, Ralf E. Wellinger

**Affiliations:** 1 Departament de Ciències Mèdiques Bàsiques, Universitat de Lleida, IRBLleida, Rovira Roure 80, 25198 Lleida, Spain.; 2 Centro Andaluz de Biología Molecular y Medicina Regenerativa (CABIMER), Universidad de Sevilla, 41092 Sevilla, Spain.

**Keywords:** selenium, yeast, DNA damage, oxidative stress, mitochondrial function, signal transduction

## Abstract

Inorganic Se forms such as selenate or selenite (the two more abundant forms in nature) can be toxic in *Saccharomyces cerevisiae* cells, which constitute an adequate model to study such toxicity at the molecular level and the functions participating in protection against Se compounds. Those Se forms enter the yeast cell through other oxyanion transporters. Once inside the cell, inorganic Se forms may be converted into selenide through a reductive pathway that in physiological conditions involves reduced glutathione with its consequent oxidation into diglutathione and alteration of the cellular redox buffering capacity. Selenide can subsequently be converted by molecular oxygen into elemental Se, with production of superoxide anions and other reactive oxygen species. Overall, these events result in DNA damage and dose-dependent reversible or irreversible protein oxidation, although additional oxidation of other cellular macromolecules cannot be discarded. Stress-adaptation pathways are essential for efficient Se detoxification, while activation of DNA damage checkpoint and repair pathways protects against Se-mediated genotoxicity. We propose that yeast may be used to improve our knowledge on the impact of Se on metal homeostasis, the identification of Se-targets at the DNA and protein levels, and to gain more insights into the mechanism of Se-mediated apoptosis.

## INTRODUCTION

Selenium (Se) is an element that shares characteristics of both metals and nonmetals, being therefore considered as a metaloid. Similarly to sulfur, Se has different oxidation states ranging from +VI to -II. In the external environments found on earth it is present in low amounts, generally as selenate Se(+VI) or selenite Se(+IV), although these amounts vary considerably depending on the geographical areas 1. Se is an essential nutritional supplement in the human diet, and intake doses between 30 and 55 µg per day are recommended, while doses lower than 10 µg per day can be detrimental for human health 2. Thus, Se deficiency has been associated with increased risk of mortality, poor immune function and cognitive decline. This nutritional requirement is explained by the fact that Se (in the form of selenocysteine) is a component of about 25 human selenoproteins, among them several glutathione peroxidases and thioredoxin reductases 3. These two enzyme activities participate in the defense against oxidants, with a selenocysteine residue as part of the enzyme active site 4.

In addition to their known role in combating various forms of degenerative diseases, the impact of organoselenium compounds in cancer chemoprevention has been studied, and thus, doses up to 200-300 µg per day are proposed to protect against diverse types of cancer (prostate, colorectal and lung) on the basis of the Se antioxidant role 5,6,7,8,9. However, epidemiological analyses have associated high Se levels in the serum with cardiovascular disease, amyotrophic lateral sclerosis and diabetes as well as with carcinogenesis 10,11,12,13,14. Taken together, the narrow range between beneficial and toxic Se concentrations poses caution on the use of Se-enriched supplements in animal and human nutrition, and makes it also difficult to study Se effects in human (or other animal) cell models. While selenoproteins are found in bacteria, archaea, some algae and protozoa, vertebrates and invertebrates, they have not been reported in fungi and plants 15,16. Because *Saccharomyces cerevisiae* has no metabolic need for Se, it is an adequate organism to study the toxic effects of Se forms on cell functions 14. In recent years, diverse studies (some of them including -omic approaches) have investigated the toxicity of inorganic Se forms on yeast cells, at the molecular and/or physiological levels. Here, we provide an overview on the current knowledge on Se uptake, the impact of toxic Se on genome stability and other functions, and the activation of intracellular signaling events leading to Se detoxification and tolerance in yeast cells.

## Se COMPOUNDS IN NATURE

**Figure 1:**
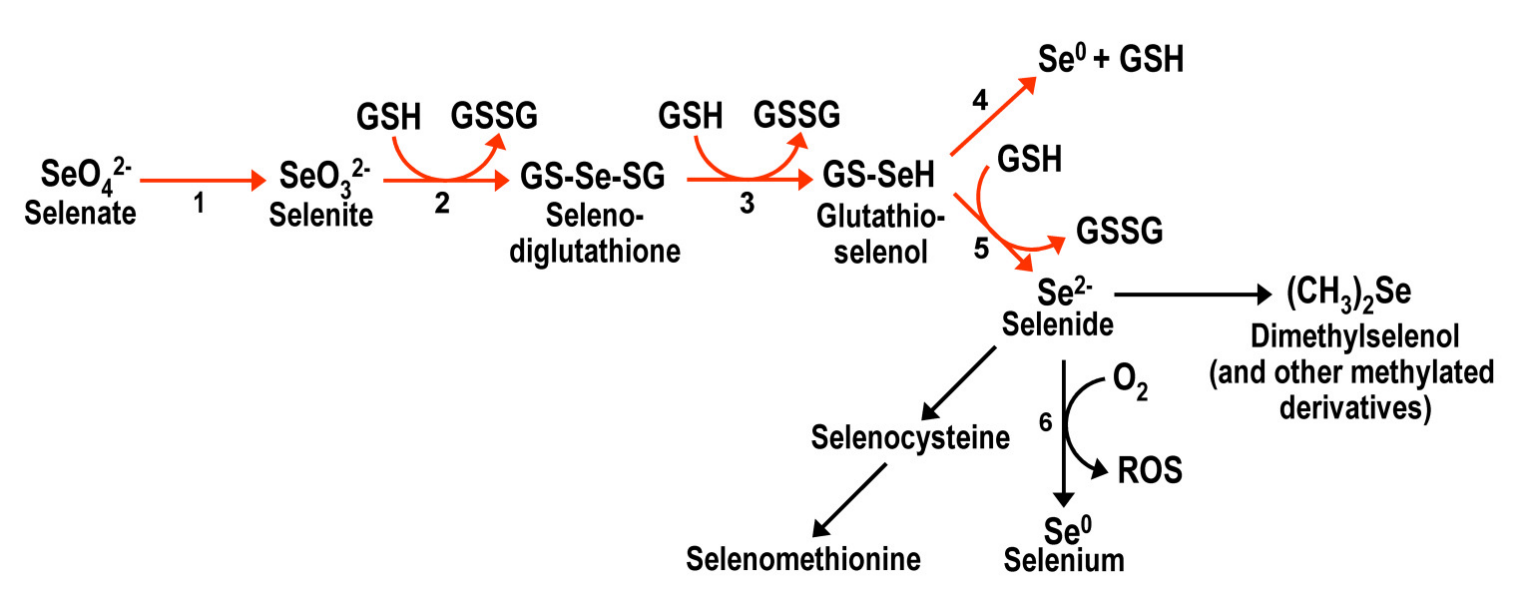
FIGURE 1: Scheme for the metabolic reduction of inorganic selenium forms, and their conversion into organic forms. Reductive reactions are indicated with red arrows. Arrow 1 corresponds to the reactions involving ATP sulfurylase and other enzymes that take part in the initial steps of the sulfate assimilation pathway. Reactions 2 to 5 are non-enzymatic and result in the net conversion of reduced glutathione (GSH) into oxidized glutathione (GSSG). Reaction 6 is also non-enzymatic and results in the formation of diverse reactive oxygen species. Adapted from 25, 26, 27 and 36.

In the environment, Se is found in four oxidation states, elemental selenium Se(0) and soluble selenate Se(+VI), selenite Se(+IV), and selenide Se(-II) (see Fig. 1). Se(-II) is found as volatile, methylated species or as organoselenium typically in the form of proteins containing the amino acids selenocysteine and selenomethionine 17. Anaerobic microorganisms such as *Thauera selenatis* can respire toxic oxyanions of Se, namely selenate and selenite 18, and reduce them to insoluble Se(0) as well as hydrogen selenide 19. These microorganisms either use selenate or selenite as their respiratory electron acceptor for the oxidation of organic carbon substrates like lactate or acetate to carbon dioxide. Thereby, these toxicants can be effectively removed from solution via a microorganism-mediated precipitation to non-toxic Se(0) 20. Thus, the common link is that Se specification in nature is strongly dependent on microbial activity 21,22. The formation of Se(0) nanoparticles (20-300 nm in diameter) by selenium-respiring bacteria and yeast is a phenomenon that deserves special mention 19,23. They occur outside the cell envelope, eventually slough off the cell surface and get released into the medium. When harvested and cleansed of their cellular parents, they were found to have curious electro-optical properties, making them candidates for further studies with “nanotechnological” applications 24.

Inorganic Se compounds can also be metabolized inside the cell through a reductive pathway from selenate to selenide (Fig. 1). This process involves reduced glutathione (GSH) with the consequent formation of oxidized glutathione (GSSG) and reactive oxygen species (ROS) 25,26,27. Selenide is also the intermediate for the formation of selenocysteine, from which selenomethionine can then be formed in organisms with a functional transsulfuration pathway. Several metabolomics studies 28,29,30 have shown that a plethora of additional organoselenium compounds accumulate in yeast cells supplemented with selenate, selenite or selenomethionine, in addition to demonstrating that selenocysteine can also be formed from selenomethionine.

## UPTAKE OF INORGANIC Se FORMS

Specific transporters for uptake of inorganic Se compounds still need to be characterized in eukaryotic cells. In *S. cerevisiae*, selenate probably enters through the high affinity sulfate permeases Sul1 and Sul2. Thus, a double mutant lacking both transporters is hyperresistant to selenate as well as to chromate 31, suggesting that both oxyanions adventitiously employ the sulfate transporters to enter into the yeast cells. Consistent with this observation, later experiments demonstrated that chromate and sulfate compete with each other to enter into the cells 32, although similar experiments have not been done with selenate. Also, heterologous expression of a plant sulfate transporter, SHST1, in *S. cerevisiae* *sul1* mutant cells allows increased uptake of molybdate, while increasing molybdate concentration in the medium interferes with sulfate entry 33, suggesting that sulfate and molybdate share the same transport system. Therefore, the Sul1/Sul2 transport system seems to be used by diverse oxyanions (+VI) for entry into *S. cerevisiae*.

Initial work on the kinetics of selenite uptake indicated the existence of both a high affinity and a low affinity transport system operating at different selenite concentrations 34. In parallel, another study pointed to the interaction between selenite and ortophosphate assimilation 35. On the other hand, reducing molecules present in the growth medium such as glutathione or other thiols would reduce selenite to hydrogen selenide, which would then be internalized into the cells to cause toxicity 36. However, there is no evidence of the existence of selenide transporters in *S. cerevisiae* or other eukaryotic organisms.

A detailed study by Lazard *et al*. 37 confirmed that selenite employs phosphate transporters to enter into yeast cells. Two different transport systems mediate ortophosphate uptake in *S. cerevisiae* 38,39. The high affinity phosphate transport system is composed of the Pho84 and Pho89 transporters and functions at both low and high phosphate concentrations. Expression of the *PHO84* and *PHO89* genes is upregulated at low phosphate concentration depending on the *PHO* signal transduction pathway, with the Pho4 transcription factor as effector of the pathway 40. Pho84 transporter operates preferentially at neutral and acidic pH, while Pho89 is functional at alkaline pH 41. The low affinity transport system operates at high phosphate concentrations, is composed by Pho87, Pho90 and Pho91, and is post-transcriptionally downregulated at low phosphate conditions by Spl2, a member of the *PHO* regulon 38,39. Depending on phosphate levels in the medium, selenite enters the yeast cell through Pho84 or Pho87/Pho90/Pho91 37. At very low phosphate levels (up to 0.1 mM) selenite enters efficiently through Pho84 and is highly toxic. Given that Pho84 displays a much higher affinity for phosphate than for selenite, while the phosphate low affinity system is considerably more unspecific, phosphate favorably competes with selenite at moderately higher phosphate levels (up to 0.4 mM) and selenite becomes less toxic. At still higher phosphate levels selenite enters through the less discriminatory low affinity system and becomes highly toxic again 37. Interestingly, arsenate also can adventitiously enter *S. cerevisiae* cells through the Pho84 and phosphate low affinity transporters 42,43, indicating that phosphate transporters contribute to the toxic uptake of a wide range of compounds 44.

Glucose was the only carbon/energy source used in the studies described above but apparently, the Jen1 transporter acts as an alternative selenite and arsenite transporter in the presence of other carbon sources 45. The *JEN1* gene codes for a plasma membrane high affinity transporter of monocarboxylic acids such as lactate, pyruvate or acetate 46, and monocarboxylic acids can compete with selenite for entrance into the yeast cell 45. Glucose represses expression of *JEN1* 47, and induces endocytosis and ubiquitin-mediated degradation of the Jen1 protein 48. Thus, the Jen1 transporter would not operate in glucose medium where the above mentioned phosphate transporters would be the mediators of selenite uptake. The efficient adventitious transport of selenite by Jen1 in conditions where a carbon source alternative to glucose is employed by the yeast cells would explain why selenite toxicity is increased in respiratory growth conditions 49,50.

In summary, selenite opportunistically employs mechanisms involved in transport and metabolic conversion of essential nutrients in order to enter into yeast cells. Interestingly, overexpression of the *SSU1* gene (encoding for a plasma membrane sulfite pump which acts as a sulfite detoxifier in yeast cells 51) also confers selenite tolerance 52, indicating the existence of common export mechanisms for sulfite and selenite.

## MECHANISMS OF Se TOXICITY IN YEAST CELLS

Selenite and selenide cause the death of *S. cerevisiae* cells in a dose-dependent manner 52,53,54. In contrast, equivalent concentrations of organic forms of Se (selenocysteine or selenomethionine) do not provoke lethality 53, although some inhibitory effects on cell growth may occur, at least in the case of selenomethionine 29,55. Selenate is also toxic for yeast cells, although considerably less than selenite when equivalent concentrations are compared for effects on cell viability, ROS production or DNA damage 56. This could be due to less efficient uptake of selenate or only partial metabolic conversion to selenite and selenide. Cells lacking the Met3 ATP sulfurylase activity are resistant to high selenate concentrations 57, yet the question if its resistance is due to impaired selenate to selenite conversion or a consequence of a repression/inhibition of sulfate transporters is still under debate 58. It would be interesting to see if overexpression of *MET3* or genes coding for related enzymatic activities may be a tool to further dissect the contribution of selenate uptake or its intracellular metabolic conversion to selenate toxicity.

### Genotoxic effects

Toxicity of the inorganic Se forms results from different physiological effects that are interrelated. Selenite-treated yeast cells are prone to DNA double strand break (DSB) formation and show increased mutation rates 52,53. DSBs may result from the chemical alteration of DNA bases by selenite-mediated ROS that challenge replication fork integrity and genome stability in proliferating cells. However, although at lower levels, selenite also provokes DSB formation in stationary phase yeast cells 53, pointing to DNA replication-independent damage mediated by selenite (or its metabolic derivative selenide, see below). Selenite compounds have been shown to induce apoptotic death of tumor cells involving topoisomerase II (Top II) 59,60. Top II action involves the cleavage of both DNA strands 61 and selenite stabilizes reversible TopII cleavage complexes *in vitro*, suggesting that the stimulation of Top II action may be a main source of selenite-mediated DNA breaks. Selenide also breaks DNA phosphodiester bonds *in vitro* under aerobic conditions, due to the action of ROS different from O2•-, and similar effects may be caused by selenite provided that GSH is present in the reaction mixture 55.

In yeast cells DNA DSBs are mainly repaired by the homologous recombination (HR) pathway, taking advantage of sister chromatids as DNA repair templates 62. In accordance, a genome-wide analysis of selenite sensitive mutants in haploid *S. cerevisiae* cells has revealed that HR mutants are selenite-hypersensitive 63, confirming previous results on the importance of HR in protecting yeast cells against selenite-induced DNA damage 52,64. In addition to HR, Rad5/Rad6-mediated post-replicative repair (PRR) is required to protect cells from selenite-mediated DNA damage 61,65. Although HR and PRR seem to have a synergistic effect in repairing selenite-mediated DNA damage, exposure to selenite does not stimulate the expression of DNA repair genes 63,66. Notably, despite the role of non-homologous end-joining (NHEJ) in the repair of DNA DSBs 67, mutants impeded in NHEJ were dispensable for tolerance to selenite-mediated DNA damage 68. NHEJ is the markedly preferred option for the repair of DNA lesions formed outside of the S/G2 phase of the cell cycle 69, suggesting a predominant impact of selenite on the genome stability of replicating and/or dividing cells.

A recent study by Peyroche *et al*. revealed that selenide treatment causes oxygen-dependent DNA phosphodiester-bond breaks *in vitro*, and chromosome fragmentation *in vivo* 54. The same study included a genome-wide screen to identify mutants that confer selenide hypersensitivity. Apparently, the repair of selenide-mediated lesions depends on HR, suggesting an overlap in the kind of DNA lesions generated by selenide and selenite. Based on these observations, it is conceivable that cells reduce selenite to selenide in the presence of oxygen thereby promoting ROS and DNA damage formation 54.

Despite the advance in our knowledge on the mechanisms that contribute to the formation and repair of Se-mediated DNA damage, little is known on the possible impact of Se on proteins involved in DNA metabolism. It will be interesting to see if Se compounds interfere with disulfide bridge formation and protein folding, or the function of metalloproteins such as DNA polymerases 70.

### Alteration of mitochondrial functions

Mitochondria play an important role in cellular energy supply and apoptosis in yeast and higher eukaryotes 71,72. Mitochondrial functions are highly conserved from yeast to human, and yeast-based assays have been employed to identify drugs active against human mitochondrial disorders 73. The intermembrane space of mitochondria contains several pro-apoptotic proteins, including cytochrome c, procaspases 2, 3, and 9, and apoptosis-inducing factor, all of which are released into the cytosol as a result either of disruption of the outer mitochondrial membrane or of the opening of specific pores 74. The opening of the mitochondrial permeability transition (MPT) pore induced by apoptotic stimuli is thus thought to result in the swelling of the mitochondrial matrix and consequent rupture of the outer membrane and release of pro-apoptotic proteins. MPT pore opening is regulated by Ca^2+^, thiol oxidants, ROS, and members of the Bcl-2 family of proteins 75,76,77,78. *S. cerevisiae* has been very useful to dissect the underlying mechanisms that contribute to apoptosis but little is known on the impact of Se on yeast mitochondrial function. In human cells, apoptotic events that are mediated by selenite, selenocystine, and selenodioxide are related to oxidation of protein thiol groups and ROS generation 79. In addition, selenite has been found to promote transitions in mitochondrial permeability, and cytochrome c release in isolated mitochondria 80. However, enhanced Se uptake has also been shown to improve mitochondrial function, most likely because 3 out of 25 mammalian selenoproteins (TR2, GPX4 and SelO) were shown to reside in mitochondria. These proteins function in the regulation of mitochondrial redox homeostasis and antioxidant activity 81,82,83. Despite the fact that *S. cerevisae* is lacking mitochondrial selenoproteins, toxic Se may as well induce apoptotic events in yeast 50. Se and mitochondrial DNA are not essential for *S. cerevisae* growth 84, thus it was possible to identify yeast genes involved in mitochondrial function that affect intracellular Se levels 85. It remains to be explored as to whether mutations in human genes related to mitochondrial function will affect mitochondrial Se levels, or be relevant in disease formation.

### Effects on the cellular redox state

Once inside the cell selenite would employ the sulfate assimilation pathway for its conversion into the more toxic form selenide 86. In fact, a genome-wide screen for *S. cerevisiae* mutants displaying selenite tolerance revealed that the mutants in genes involved in the conversion of sulfate into sulfur were tolerant to selenite and also to tellurite, supporting an opportunistic common assimilation pathway for both toxic oxyanions 86. The sulfur (or selenite) assimilation pathway involves a sequence of redox reactions 87. In the case of selenite, the above study also demonstrated that intracellular selenite reduction is linked to GSH metabolism, as a *gsh2*Δ mutant defective in GSH biosynthesis is also unable to accumulate elemental selenium 86, although this does not result in increased tolerance to selenite 63,86. The involvement of GSH in selenite reduction has also been initially shown in bacteria 88. In yeast, the Glr1 glutathione reductase is the enzyme involved in maintaining most of the glutathione intracellular pool in a reduced state. This tripeptide molecule is the main intracellular redox buffer, as it is needed for the activity of redoxins, peroxidases and metal-detoxifying enzymes among others, besides forming reversible disulfide bonds with protein thiols to protect them against irreversible oxidation 89. Selenite causes a decrease of the GSH/GSSG ratio in the cell, in addition to reduction of total glutathione 28,90,91. Accordingly, overexpression of *GLR1* partially rescues the inhibitory effect of selenite on yeast cell growth 52, while a *glr1*∆ mutant is hypersensitive to selenite, as well as a *gsh1*∆ mutant involved in the first step of GSH biosynthesis 63. The *glr1*∆ and *gsh1*∆ mutants have also been described as hypersensitive to sodium selenide 54, again pointing to common toxicity effects between both inorganic forms of Se. A drop in intracellular GSH levels should constrain the activity of GSH-dependent ROS-detoxifying enzymes, and thus lead to ROS accumulation and macromolecular damage. Accumulation of intracellular hydrogen peroxide upon selenite treatment has been reported 49, and in addition to the above described genotoxic effects, selenite also provokes irreversible ROS-mediated carbonylation of protein side chains 50. A metabolomics study has shown that the drop in intracellular GSH levels is much lower in selenate-treated cells than in selenite-treated ones 28, which could contribute to the lower toxicity of selenate.

The observed drop in the intracellular glutathione pool upon selenite treatment cannot be simply explained by the reduction of the cytosolic GSH pool due to reduction of selenite to selenide and ROS. Detoxification of heavy atoms such as cadmium, arsenic or mercury in yeast involves the participation of the vacuolar membrane located ABC transporter Ycf1 44,92. Ycf1 internalizes GSH-heavy metal conjugates into the vacuolar lumen, therefore conferring heavy metal tolerance and thus, the *ycf1*∆ mutant is hypersensitive to heavy metals. On the contrary, the *ycf1*∆ mutant is resistant to selenite, while overexpression of Ycf1 exacerbates selenite toxicity 90, indicating a more complex function of the Ycf1 pump in selenite tolerance. Ycf1 is able to transport GSSG and selenodiglutathione (GSSeSG) 90. This finding led to the proposal of a vicious cycle of selenite-mediated cytosolic GSH depletion, based on a continuous Ycf1-dependent vacuolar internalization of GSSG and GSSeSG. In the vacuole, GSSeSG converts to GSSG and selenide. In this case, selenide would again diffuse to the cytosol, while GSSG would be retained in the vacuole causing cytosolic glutathione depletion. Such scenario explains how vacuolar internalization of Se compounds alters the intracellular redox state. However, the functional integrity of vacuoles is also essential to cope with Se toxicity 63, as it will be discussed below.

Transcriptome analysis of selenide- and selenite-treated *S. cerevisiae* cells revealed the induction of genes participating in the oxidative stress response 54,63,65 that are expressed under control of the AP-1 family like Yap1 transcription factor 93. Genes that were upregulated by selenite included thioredoxin reductase (*TTR1*) and glutathione reductase (*GLR1*), both coding for activities required for functional (NADPH-dependent) thioredoxin and (glutathione-dependent) glutaredoxin systems, respectively. These two systems control the redox state of protein thiol groups through their thiol oxidoreductase activity and consequently, are important for repairing oxidation of these thiol groups and for protein redox modulation 94,95. Selenite also induces expression of peroxidases, the cytosolic catalase and Cu/Zn-dependent superoxide dismutase *SOD1* 66. Because these enzymes are involved in ROS detoxification, these observations reinforce the idea that the selenite/selenide treatment of the yeast cells causes alterations in the intracellular redox state.

The alteration of the GSH/GSSG ratio by selenite may result in general oxidation of protein thiol groups. This would explain the reported protection of dithiol glutaredoxins Grx1 and Grx2 against selenite toxicity 49,50. In fact, Grx1 and Grx2 have a defense function against oxidative stress in *S. cerevisiae* 96, although the biochemical bases of such differential protection are not characterized. A double *grx1*∆ *grx2*∆ mutant is hypersensitive to selenite 49,50, while the respective single mutants are not, which would support overlapping roles for both glutaredoxins during selenite stress 50. Another study, however, has attributed a more important role to Grx1 in such protection, relating it to the predominant participation of superoxide in selenite-generated oxidative stress 63. The *grx1*∆ mutant has also been described as selenide-hypersensitive 54. All these studies commonly point to the importance of yeast dithiol glutaredoxins in protection against selenite and selenide, probably through regulation of protein thiol oxidation. The reported peroxidase activity of those glutaredoxins 97 might as well contribute to such protection.

## SIGNALING PATHWAYS FOR Se STRESS

Diverse stress response pathways sense the lesions caused by Se compounds in yeast cells and induce protective responses. Given the diversity of the toxic effects triggered by selenite or selenide, it is not surprising that several response transducers and effectors may be acting in parallel. Disruption of the pathways or effector loss of function results in hypersensitivity to Se molecules.

### The DNA damage checkpoint pathway

Upon DNA damage, cell cycle arrest by G1/S, S and G2/M cell cycle checkpoints is essential to avoid unscheduled repair of DNA lesions 98. In yeast, Rad9 is a central mediator of checkpoint activation throughout the cell cycle 99. DNA lesions induce cyclin-dependent kinase or Mec1 mediated Rad9 phosphorylation and subsequent activation of signal transducers such as Rad53, which itself phosphorylates diverse downstream effectors 100,101,102. In addition, Mec1 accumulation at stalled replication forks activates Mrc1, a replication fork component needed to upregulate Rad53 phosphorylation 103. Therefore, several phosphorylation events could act in parallel to promote the activation of DNA damage response mediator proteins. Mutants in *RAD9* and other genes of the pathway are hypersensitive to DNA damage mediated by UV light, DNA alkylating agents or selenite 54,99. Because selenite treatment causes cells to arrest at G2/M 52, Rad9-dependent activation of the DNA damage checkpoint pathway seems to be important for the selenite-dependent cell cycle arrest. Furthermore, checkpoint mutants with a specific function in different stages of cell cycle are hypersensitive to selenite 52,65, suggesting that selenite causes DNA damage throughout the cell cycle. In contrast, mutants in *RAD9* and other genes coding for central components of the DNA damage response are not hypersensitive to selenomethionine 65. It is therefore unlikely that this organic form of Se contributes to Se-mediated genotoxicity 56.

### The Snf1 kinase pathway in response to oxidative stress

Yeast Snf1 was identified as having a general role in the oxidative stress response and selenite tolerance 91. Snf1 is a yeast member of the AMP-activated protein kinase (AMPK) family constituted by protein complexes that participate in metabolic stress responses responsible for the maintenance of cellular ATP levels in eukaryotes. Thus, Snf1 plays a key role in the adaptation of yeast cells to glucose limitation and the usage of alternative carbon sources 104. To carry out this function, upstream acting protein kinases (Sak1, Elm1 or Tos1) sense the carbon source stress conditions, phosphorylate Snf1 (with Sak1 being the major player in the response to glucose scarcity) and promote its internalization to the nucleus to activate several transcription regulator targets acting as effectors of this kinase. More recently, it has been demonstrated that Snf1 does not only regulate nuclear targets, but also can modulate the function of cytosolic proteins, such as the arrestin-related protein Rod1, which coordinates endocytosis of alternative carbon source transporters in response to glucose presence in the medium 105. The work of Pérez-Sampietro *et al*. 91 demonstrated that Elm1-dependent activation of Snf1 is required for protection against oxidants (among them selenite) causing alteration of glutathione redox homeostasis towards a more oxidized state. This protection does not require the nuclear targets of Snf1 operating during the glucose depletion response, overall defining a previously uncharacterized response against oxidative stress conditions with the participation of the Snf1 kinase. Similarly, Snf1 activity is required for cadmium tolerance independent of its nuclear targets 92. Interestingly, in human cell lines, hydrogen peroxide activates AMPK as part of a protective signaling mechanism mediated by mTORC1 106. In another study on human colon cancer cells, selenate provoked a late activation of AMPK through ROS formation, and this AMPK activation was essential to inhibit cell proliferation by downregulating the COX2-mediated pathway 107. An additional work with human cell lines also demonstrated activation of AMPK by redox changes in the α and β subunits of the AMPK complex induced by hydrogen peroxide 108. In summary, there is experimental evidence from a diversity of cell types supporting the involvement of AMPK in the response to stress by redox-altering agents, including Se compounds.

### The Rim101-mediated pathway and vacuolar functions

Rim101 is a member of the fungal PacC family of C_2_H_2_ zinc finger transcriptional regulators that was initially characterized as modulator of the response of yeast cells to alkaline pH. Later studies showed its implication in processes such as sporulation and invasive growth, protection against Na^+^ and Li^+^ toxicity, cell wall assembly, protection against weak organic acids and regulation of calcium homeostasis 109,110,111,112. A recent study 113 has extended the range of cell processes regulated by Rim101 to vacuolar functions. In this process Rim101 would act cooperatively with the ESCRT complex, a protein complex that was originally identified as being important for the sorting of ubiquitinated endosomal membrane proteins into the multivesicular body (MVB) 114,115. Further studies demonstrated additional roles of the ESCRT machinery in other cellular processes 114,115, including the Rim101 signaling pathway in yeast 110,111. In the absence of Rim101, expression of several VMA genes implicated in the synthesis of subunits of the vacuolar H^+^-ATPase (V-ATPase) complex becomes downregulated, providing a rationale for the selenite hypersensitivity of the rim101∆ mutant 113. On the contrary, constitutive activation of Rim101 prevents inhibition of vacuolar acidification caused by selenite. V-ATPase activity is required to maintain the acidity of the vacuolar lumen necessary for importing a number of different molecules into the vacuole, including the toxic ones 116. These observations, together with the fact that mutants in the genes encoding the different V-ATPase subunits are hypersensitive to selenite 63, point to a scenario in which Rim101 modulates the vacuolar acidity necessary for selenite detoxification through its transcriptional activity. In this scenario, the ESCRT machinery would participate in maintenance of vacuolar acidity through both Rim101-dependent and -independent pathways. Consistently with this, ESCRT mutants are also hypersensitive to selenite 113.

## CONCLUSIONS AND PERSPECTIVES

**Figure 2:**
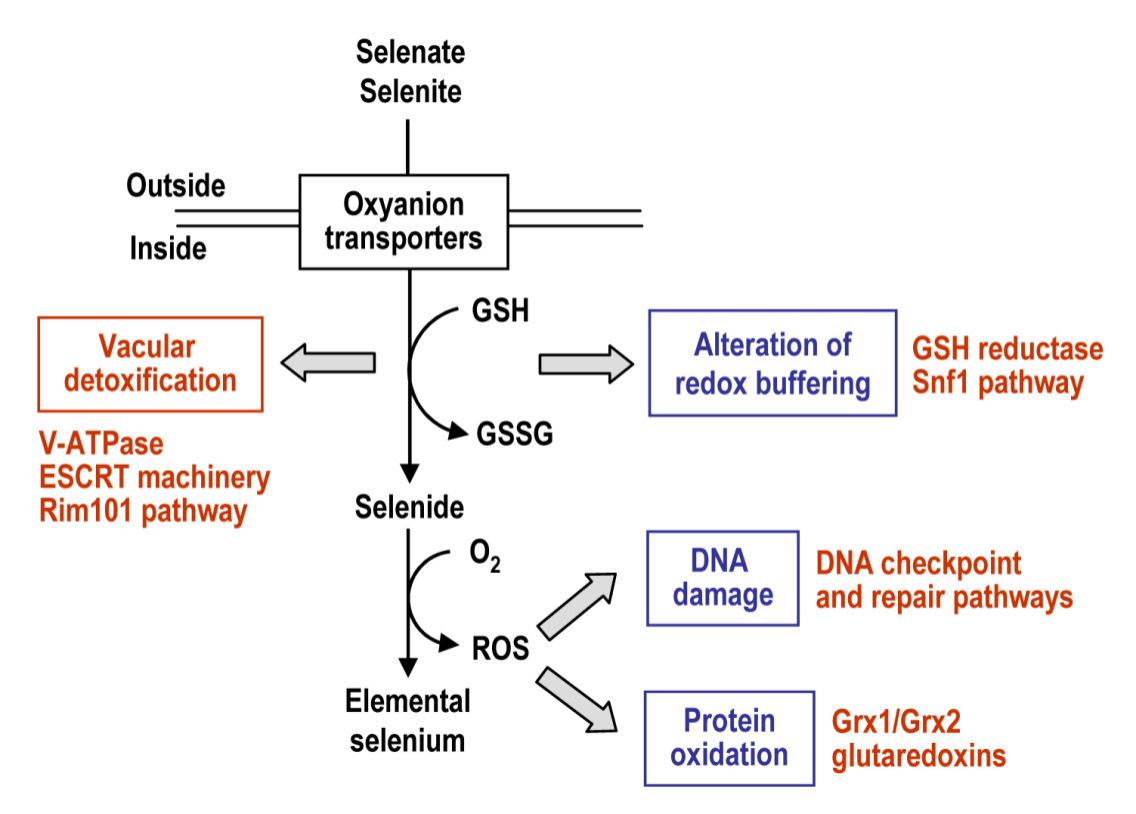
FIGURE 2: Description of factors and pathways involved in the uptake, intracellular selenium tolerance and detoxification in *S. cerevisiae*. See the text for details. Toxic consequences (blue) and protective mechanisms (red) are indicated.

The mechanisms by which Se compounds enter into *S. cerevisiae* cells and interfere with cellular processes are summarized in Fig. 2, which also depicts the activation of pathways required for Se tolerance and detoxification. Entrance of selenate and selenite (the two more abundant free forms of Se in nature) occurs through oxyanions transporters, and once in the cell they are transformed into selenide through a reductive pathway that may involve GSH. In the presence of oxygen, selenide can promote the formation of superoxide and other ROS species, which may damage DNA, proteins and probably also other cellular macromolecules. Thus, changes in the redox buffering of the cell and ROS overproduction, which themselves are two interrelated processes, would be an origin of Se toxicity.

However, many questions remain. Selenite has been shown to activate expression of genes under transcriptional control of the Aft1 regulon 66. Genes affected encode for proteins required for the high-affinity uptake of iron and redistribution of internal iron stores under iron scarcity 117,118. In addition, cells lacking Aft1 are moderately hypersensitive to selenite, this phenotype being rescued by iron supplementation to the growth medium 113. These observations may indicate that selenite (or selenide) would interfere with iron bioavailability, the Aft1-mediated response being required in such conditions. A study has addressed the ability of selenide to form insoluble complexes *in vitro* with different metal anions, confirming in fact its interaction with ferrous iron and that the selenide- iron complexes become biologically inactive 119. The possibility that *in vivo* interference can be extended to other anions is open. In addition, yeast as model organism offers the possibility to address the identification of hot spots of Se-mediated DNA damage, or to extend our knowledge on factors involved in signaling and repair of Se-mediated damage. It will be interesting to determine which proteins are prone to selenite or selenide-dependent modifications, including irreversible (i.e. carbonylation) or reversible (phosphorylation, ubiquitination, sumolyation) protein modifications. Finally, yeast may serve as an excellent tool for the characterization of events related to Se-mediated aging and apoptosis. Such studies are important for a better understanding of the molecular mechanism underlying Se-mediated pathologies in multicellular organisms, including humans.
